# Aqueous extract of *Sanguisorba officinalis* blocks the Wnt/β-catenin signaling pathway in colorectal cancer cells†

**DOI:** 10.1039/c8ra00438b

**Published:** 2018-03-13

**Authors:** Meng-ping Liu, Wa Li, Cong Dai, Christopher Wai Kei Lam, Zheng Li, Jie-feng Chen, Zuan-guang Chen, Wei Zhang, Mei-cun Yao

**Affiliations:** School of Pharmaceutical Sciences, Sun Yat-sen University Guangzhou 510006 P. R. China yaomeicun@gmail.com; State Key Laboratory of Quality Research in Chinese Medicine, Macau Institute for Applied Research in Medicine and Health, Macau University of Science and Technology Taipa Macau China wzhang@must.edu.mo

## Abstract

*Sanguisorba officinalis* (the Chinese name is DiYu, DY) exerts significant anti-proliferative activities against colorectal cancer (CRC) cells. Since most of CRC result from the aberrant activation of the Wnt/β-catenin signaling pathway, inhibitors of the Wnt pathway are considered as promising anti-CRC agents. This study aimed to investigate whether DY could be a potential herbal Wnt inhibitor, and the bioactive constituents and underlying molecular mechanisms for DY's inhibiting activities would be studied as well. Accordingly, the inhibitory activities of DY and its main components against the Wnt pathway were assessed using the single-luciferase reporter assay based on HEK293 cells. Additionally, the levels of key Wnt-related genes or proteins were measured to verify the inhibitory effects on the Wnt pathway of CRC cells. Finally, the underlying mechanisms accounting for the efficacy of candidate drugs were explored by the transcriptomic study. Results show that DY and its tannins (RZ), and saponins (ZG) significantly inhibited the Wnt pathway of HEK293 cells activated by wnt3a. However, their respective constituents were not effective as expected. Additionally, DY and RZ prominently down-regulated the levels of β-catenin and Wnt-targeted genes including *Axin2*, *c-Myc* or *CyclinD1* of three CRC cells. Transcriptomic profiling study suggests that the down-regulation of the mRNA levels of Wnt-related genes such as *LPAR6* may be associated with the inhibitory effects of DY and RZ on the Wnt pathway of HT29 cells. Therefore, our studies first uncovered the blocking activity of DY on the Wnt pathway, providing evidence for the rationale of developing Wnt inhibitors from DY as anti-CRC agents.

## Abstract 2

*Sanguisorba officinalis* (the Chinese name is DiYu, DY) exerts significant anti-proliferative activities against colorectal cancer (CRC) cells.

## Graphical abstract

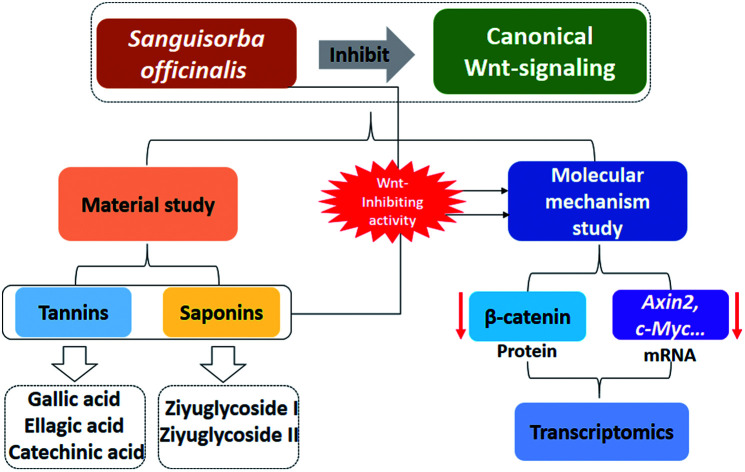

## 1. Introduction

As the third most common carcinoma worldwide, colorectal cancer (CRC) seriously threatens human health.^1^

The pathogenesis of CRC is complex and is still not clearly understood. However, based on current reports, the aberrant activation of the Wnt/β-catenin pathway is believed to be a major factor contributing to the progress of CRC.^2^ In this signaling, β-catenin is a key functional protein that's stabilized by a destruction complex. Proteins, namely *APC*, *Axin*, *CK1* and *GSK3β*, are the main components of this complex, which cooperate with each other to avoid activating the Wnt pathway by degrading the cytoplasmic β-catenin.^3^ Mutation of the tumour-suppressor genes or oncogenes such as *APC*, *Axin2*, *SMAD4* or *KRAS* would cause degradation of the complex and allows β-catenin accumulated in the cytoplasm to transfer into the cell nucleus. Then Wnt/β-catenin signaling is prone to be activated when nucleus β-catenin integrating with TCF/LEF transcription factors.^4,5^ Subsequently, through activation of the TCF encoding genes, Wnt pathway activators would result in the progression and metastasis of CRC.^6^ Therefore agents exerting inhibitory activities towards Wnt/β-catenin pathways are potentially anti-CRC medicines. Up to now, a great number of Wnt signaling inhibitors have been discovered, most of which are small molecule compounds or antibodies. Although some of them exhibit significant inhibitory effects (IC_50 LGK947_ = 0.4 nM),^7,8^ their cytotoxicity remains a critical issue to be concerned.^9^

In addition to western medicines, Chinese medicines have also achieved satisfactory efficiencies in treating human cancers. Compared to the western medicine, Chinese medicines have lower cytotoxicity and much more therapeutic targets, enabling them to be a better source of anti-CRC agents. Therefore, herbal Wnt inhibitors are also worthy being developed as potential anti-CRC therapeutics. Recent studies reported that some traditional Chinese medicines such as ginseng,^10^ white hellebore,^11^*Tripterygium wilfordii*^12^ and *Coptis chinensis*^13^ were found to exert potential anti-CRC activities. However, as the inhibitory effects of these herbs against the Wnt pathway were not strong enough,^14^ their anti-cancer effects were unsatisfactory, indicating that it is important to screen and discover new herbal Wnt inhibitors with higher inhibitory activities for clinical use.

*Sanguisorba officinalis* is a traditional Chinese medicine that has been widely used in the folk for treating gastrointestinal diseases. Our previous study found that a water extract of DY exhibited strong antiproliferative activities against CRC cells. Based on the close association of CRC with the Wnt/β-catenin pathway, we hypothesized that DY may exhibit an inhibitory effect towards Wnt signaling. Therefore, the aims of this study are to verify the inhibitory effect of DY against the Wnt pathway and to investigate the underlying mechanism. In addition, the potential active component of DY that's responsible for the inhibition will be studied as well.

## 2. Materials and methods

### 2.1 Plant materials

DY is widely planted around China. We only selected its genuine medicinal materials for our study. The origins and voucher numbers of the 7 batches of *Sanguisorba officinalis* L. radix (DY, D1–D7) are listed in Table s1.† All of them were authenticated by Prof. Depo Yang (Sun Yat-sen Univerisity) and stored in the Lab of Pharmaceutical Analysis and Quality Assessment, School of Pharmaceutical Sciences, Sun Yat-sen University, China. Preparation of the total DY extracts, tannins (RZ) and saponin (ZG) fractions, and the corresponding HPLC analysis were performed as previously described.^15^

### 2.2 Chemicals and reagents

Gallic acid (GA, 98%), ellagic acid (EA, 98%) and catechinic acid (CA, 98%) were from Chengdu PUSH Bio-technology Co., Ltd. (Chengdu, Sichuan, China). Ziyuglycoside I (98%) and ziyuglycoside II (98%) were purchased from Chengdu PureChem-Standard Co., Ltd (Chengdu, Sichuan, China). DEPC solution obtained from Sangon Biotech (Shanghai, China). Wnt3a protein was purchased from EMPOWERING STEM CELL R&D (Burlingame, CA, USA). The CCK-8 kit was obtained from Dojindo (Mashikimachi, Kamimashiki Gun Kumamoto, Japan) and Steady-Glo® Luciferase Assay System was from Promega Corporation (Madison, WI, USA). PrimeSCript™ RT reagent Kit with gDNA Eraser and SYBR® Premix Ex Taq™ II (Tli RNaseH Plus) were purchased from Takara Bio Inc (Kusatsu, Shiga, Japan). RIPA reagent, Anti-β-catenin (#19807) and anti-β-actin (#4970) were from Cell Signaling Technology, Inc. (Danvers, MA, USA). Alexa Fluor 594 AFFINIpure Goat Anti-Rabbit IgG (H + L) and DAPI Fluoromount-Gtm were obtained from YEASEN Co., Ltd (Shanghai, China).

### 2.3 Cell culture

To first evaluate the activity of DY on the Wnt/β-catenin pathway, we used a stable TCF reporter cell line based on HEK293 cells obtained from Curegenix Inc. (Guangzhou, Guangdong, China). And HT29 human colorectal cancer cells were kindly gifted by Prof. Qing Li (Sun Yat-sen University, China). Both cell lines were cultured in DMEM containing 1% penicillin–streptomycin and 10% fetal bovine serum (Thermo Fisher Scientific, Waltham, MA, USA) in the incubator at 37 °C in 5% CO_2_.

### 2.4 Cell viability

HEK293 and HT29 cells were seeded in 96-well plates (10^4^ cells per well) for 24 h, which were then treated with DMEM medium supplemented with the water solution of DY or its constituents. After 24 h, cells were processed by CCK8 reagents (10 μl) according to the instructions of commercial kit and their viabilities were measured on a microplate reader (Moleculardevices, Flex Station 3, Sunnyvale, CA, USA) at 450 nm.

### 2.5 Luciferase reporter assay

The HEK293 cells were seeded in the 96-well plates at the density of 2 × 10^4^ cells per well for 24 h. Then cells were co-treated with wnt3a (100 ng ml^−1^) and DMEM medium with DY or its constituents for another 24 h. After that, cells were cultured with Steady-Glo^@^ assay reagent (30 μl per well) on the orbital shaker plate for 5 min. The mixed solutions were accordingly transferred to the HTS Transwell®-96 Receiver plates (Corning, NY, USA), and Monochromators Based Multimode Microplate Reader (Tecan, M1000 EVO75 8 plus 1, Männedorf, Switzerland) was loaded to detect the activities of the Wnt-based luciferase.

### 2.6 Western blotting assay

Cells (6-well plate, 4 × 10^5^ cells per well) were respectively treated with wnt3a (control group), DY + wnt3a, RZ + wnt3a and ZG + wnt3a for 24 h, which were then digested by trypsin for 10 seconds and washed twice with PBS solution. By centrifuging 3 min at 3000 rpm, the cell pellets were incubated with 100 μl RIPA reagent on ice for 30 min to obtain the cellular lysates. The lysates were centrifuged at 10 000*g* for 20 min at 4 °C. The supernatant was collected as the total protein extract, the concentration of which was analyzed using the bicinchoninic acid (BCA) method. 30 μg of protein sample was loaded on 8% gel to perform the sodium dodecyl sulphate-polyacrylamide gel electrophoresis, which was then transferred to PVDF membranes (Millipore, Darmstadt, Germany). After blocking with 5% skim milk powder solution (2 h), the transfected films were blotted with the anti-β-catenin and anti-β-actin for 2 h, following by 1 h of HRP-conjugated anti-rabbit secondary antibody incubation. Finally, the targeted protein bands were visualized and imaged by the ChemiDoc XRS+ system (Bio-Rad, Hercules, CA, USA).

### 2.7 Total RNA extraction and reverse-transcription-quantitative polymerase chain reaction (RT-qPCR)

Cells were treated in the same way as described in western blotting assay. After drug treatment, total RNA from cell samples were extracted. In brief, each sample was first lysed by 0.5 ml Trizol reagent (Invitrogen, USA), and then 100 μl chloroform was added followed by one-minute vortex. Collected the supernatants after 10 minutes centrifugation at 12 000*g*, which were then added to 600 ml isopropanol followed by 10 minutes centrifugation. Discarded the supernatants and added 1 ml pre-cooling 75% ethanol. Gently inverted the samples for 10–20 times and centrifuged them to collect the cell pellets, which were then dissolved in 30 μl DEPC water. Their concentrations were measured by the Qubit2.0 (Thermo Fisher Scientific, Waltham, MA, USA) at 260/280 nm. Subsequently, 10 μg total RNA was used to synthesize the complementary DNA (cDNA) based on the manufacturer's instructions of PrimeSCript™ RT reagent Kit. In brief, the gDNA of the RNA samples was first eliminated using 2 μl gDNA eraser buffer and 11 μl gDNA eraser. 10 μl of the gDNA-free RNA was then added into the reverse-transcription system (1 μl PrimeScript RT Enzyme Mix I, 1 μl RT Primer Mix, 4 μl PrimeScript Buffer2, 4 μl and RNase Free dH_2_O) to produce the cDNA (37 °C, 15 min; 85 °C, 5 s). With 2 μl cDNA (<100 ng), other reaction reagents in the SYBR® Premix Ex Taq™ II (Tli RNaseH Plus) Kit including 12.5 μl SYBR Premix Ex Tap II, 1 μl PCR Forward/Reverse Primer and 8.5 μl dH_2_O were mixed according to the kit's instructions. The primers sequences used were: *Axin2*, forward, 5′-AGGCTAGCTGAGGTGT-3′, and reverse, 5′-AGGCTTGGATTGGAGAA-3′; DKK-1, forward, 5′-CTGCAAAAATGGAATATGTGT-3′, and reverse, 5′-CTTCTTGTCCTTTGGTGTGA-3′; *c-Myc*, forward, 5′-CCACACATCAGCACAACTACG-3′, and reverser, 5′-CCGCAACAAGTCCTCTTCAG-3′; *CyclinD1*, forward, 5′-TCGGGAGAGGATTAGGTTCC-3′, and reverse, 5′-GTCACTGGATGGTTTGTTGG-3′; *FGF20*, forward, 5′-ATTCATCAGTGTGGCAGTGG-3′, and reverse, 5′-GCTCCCTAAAGATGCATTCG-3′; *GAPDH*, forward, 5′-AGGTCGGAGTCAACGGATTTG-3′, and reverse, 5′-TGTAAACCATGTAGTTGAGGTCA-3′.

For the procedures of qPCR please refer to the methods described in Bae's, *et al.*^16^ Briefly, the amplification conditions were 30 s at 95 °C, 40 cycles of 5 s at 95 °C and 30 s at 60 °C, which was performed on Bio-Rad PCR system (Bio-Rad, CFX96, USA). The obtained data were analyzed by the ΔΔ*C*_t_ approach.

### 2.8 Immunofluorescence assay

Cells (15 mm confocal dishes, 6 × 10^4^ cells per well) were treated with DY, RZ or ZG for 24 h, which were then washed once with PBS solution. The cells were fixed with 4% paraformaldehyde for 30 minutes. Then the cells were washed in PBS solution and permeabilized with 0.1% Triton X-100. Blocked in 5% skim milk solution (30 min), the cells were blotted with anti-β-catenin and anti-β-actin for 2 h, after cells incubating with Alexa Fluor 594 AffiniPure Goat Anti-Rabbit IgG (H + L) for 1 h, they were washed with PBS and then mounted with DAPI Fluoromount-G™. Finally, the cells were observed and imaged by the confocal microscopy (Zeiss, Smartproof 5, Germany).

### 2.9 The transcriptomics analysis

Prior to the sequencing analysis, HT29 cells (6-well plate, 4 × 10^5^ cells per well) were treated DY, RZ and ZG solutions for 24 h. The samples were then washed and lysed by 1 ml TRizol reagent. The transcriptomics analysis was accomplished by our collaborator, Biomarker Technologies (Beijing, China). The analytic steps were as follows: at first, 1% agarose gels were used to monitor RNA degradation and contamination. RNA purity was assessed on NanoPhotometer spectrophotometer (IMPLEN, CA, USA). And the concentration and integrity of RNA were respectively measured by Qubit RNA Assay Kit in Qubit 2.0 Fluorometer (Life Technologies, CA, USA) and RNA Nano 6000 Assay Kit of the Agilent Bioanalyzer 2100 system (Agilent Technologies, CA, USA). Then, RNA sequencing samples were prepared based on 1 μg RNA per sample. NEBNext UltraTM RNA Library Prep Kit for Illumina (NEB, USA) was used to establish sequencing libraries. Briefly, mRNA was purified from total RNA using poly-T oligo-attached magnetic beads, which was then fragmented using divalent cations. First strand cDNA was synthesized by using random hexamers primer and M-MuLV Reverse Transcriptase (RNase H-), and the second one was then synthesized by DNA polymerase I and RNase H. After a further modification of the synthesized cDNA, PCR was performed and their products were purified and assessed on the Agilent Bio-analyzer 2000 system. Afterwards, based on the established library, an Illumina Hiseq 2500 platform was applied to conduct the sequencing, and paired-end reads were generated. The depth of sequencing coverage was 10× and the length of sequence reads was from 200–250. In this study, at least 6 Gb clean data were obtained for each sample. Before data analysis, raw data were processed by removing reads containing adapter, ploy-N and low quality. Only the clean data with a high match (the quality score of the bases reached to Q30) or only one mismatch was further analyzed and annotated based on the reference genome (GRCH38) were subjected to further analysis by Tophat2 tools software. Differential expression analysis (without biological replicates) of two samples was conducted on the DEGseq (2010) R package. *Q* value < 0.005 and |log 2 (fold change)| ≥ 1 was set as the threshold for significantly different expression. GO enrichment analysis of DEGs was performed on GOseq R packages (http://www.geneontology.org/) and KEGG pathway analysis of DEGs was implemented by the KOBAS 3.0 software.

### 2.10 Statistics analysis

In addition to the transcriptomics study, all experiments have repeated thrice and their results were expressed as mean ± SD. For the statistical analysis, one-way ANOVA approach based on Kruskal–Wallis test was performed, which was followed by *post hoc* test. For the data violating homoscedasticity, data transformation was first conducted before ANOVA analysis. Statistics work was performed on the SPSS 18.0 software (SPSS Inc., Chicago, IL, USA). *P* < 0.05 demonstrated a statistically significant difference.

## 3. Results and discussion

### 3.1 DY and its active constituents inhibited the Wnt/β-catenin pathway in HEK293 cells

**Fig. 1:**
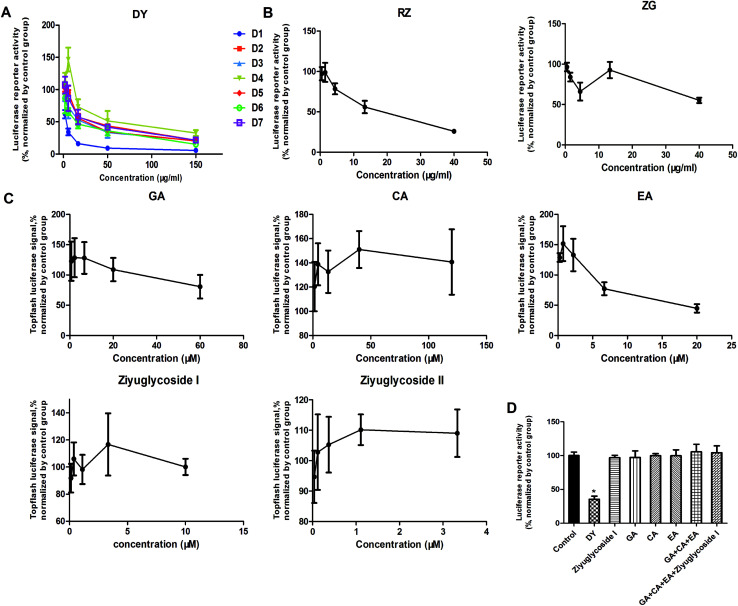
Inhibitory effects of DY and its main constituents on the Wnt/β-catenin signaling pathway. (A) Inhibiting activities of 7 batches of DY on the Wnt pathway. (B–C) The effects of the main constituents in the aqueous extract of DY (D1), namely RZ, ZG and their respective monomers (GA, CA, EA, ziyuglycoside I and ziyuglycoside II), on the Wnt pathway. (D) The influences of 15 μg ml^−1^ DY (D1) and its single/combined monomer on the Wnt/β-catenin pathway. The concentrations of ziyuglycoside I, GA, CA and EA were 0.27 μg ml^−1^, 0.28 μg ml^−1^, 0.25 μg ml^−1^ and 0.18 μg ml^−1^, respectively. *P < 0.05, indicating a significant difference was observed when compared with control group.

The HPLC results of the water extracts of D1–D7 are shown in Fig. s1A,† and the structures of two main components (GA and EA) inside identified before^15^ were listed in Fig. s1B† for further identifying the authenticity of the extract. To evaluate the modulation of Wnt/β-catenin signaling by DY extracts and its constituents, we performed a luciferase reporter assay on HEK293 cell line. To avoid any impacts of cell viabilities on the results of Wnt-based luciferase reporter assay, the concentrations of DY extracts or its components were controlled by their inhibitory percentages of viability of HEK293 cells from −10% to 10%. The results show that all the DY water extracts blocked the activities of Wnt/β-catenin signaling in HEK293 cells (Fig. 1A). Since DY from Jiangsu province (D1) exerted the most significant inhibitory effects on the Wnt pathway with an IC_50_ value at 2.20 ± 0.74 μg ml^−1^ (Table s2†), D1 was used for subsequent studies. To find out the potential active constituents in DY, we investigated the anti-Wnt effects of tannins (RZ) and saponins (ZG), which respectively take proportions of around 17% and 4% of total contents in DY,^17,18^ as well as their representative constituents, namely GA, CA, EA, ziyuglycoside I and ziyuglycoside II.^15,19^ Results indicate that RZ and ZG inhibited Wnt signaling in the concentration range of 0.5–40 μg ml^−1^ (Fig. 1B), and RZ's inhibitory effects were stronger than ZG's. However, their effectiveness was weaker than the overall water extract of DY, demonstrating that other constituents, not limited to those in RZ and ZG, were also likely to be involved in the Wnt-inhibiting activity of DY, which would be further confirmed by our study. For the representative chemicals in RZ and ZG, except for EA, GA, CA, ziyuglycoside I and ziyuglycoside II didn't exhibit any inhibitory effects. On the contrary, even up-regulated the activities of Wnt pathway (Fig. 1C). Furthermore, considering the integrality of the DY extract, we evaluated the effects of 15 μg ml^−1^ DY and its representative chemicals on Wnt pathway. 15 μg ml^−1^ was chosen was due to the inhibitory effects of DY was strong enough and most of the components inside were detectable at this concentration. Fig. 1D indicates that the combination regimen of GA, CA, EA and ziyuglycoside I at their concentrations in 15 μg ml^−1^ DY also did not show any obvious inhibiting effects on Wnt signaling, suggesting that the inhibitory activities of RZ and ZG were not mainly come from the indicated components.

**Fig. 2:**
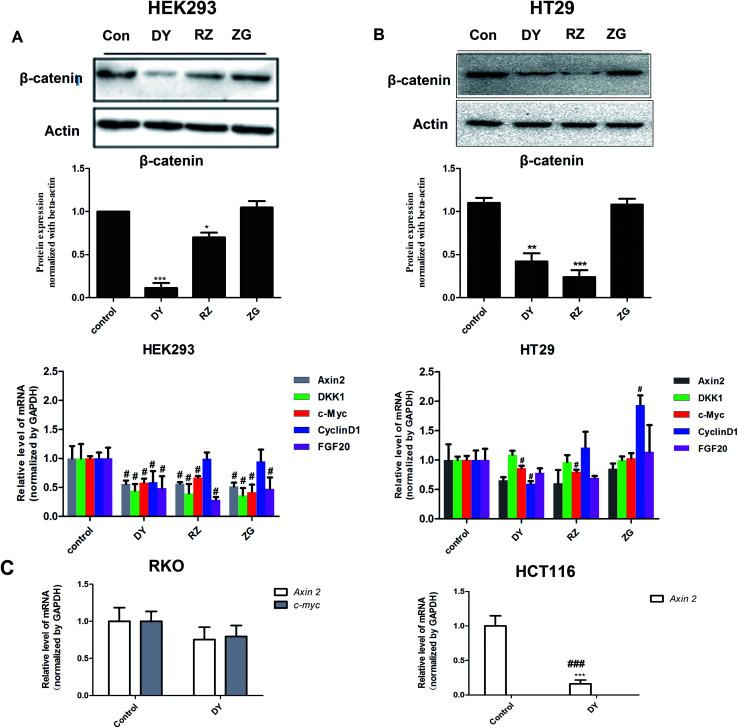
Effects of DY, RZ and ZG on the levels of key protein or genes of Wnt pathway of HEK293 cells. (A) and (B) respectively indicate that DY (30 μg ml^−1^), RZ (40 μg ml^−1^) and ZG (30 μg ml^−1^) down-regulated the levels of β-catenin protein and mRNA levels of Wnt targeted genes (*Axin2*, *DKK1*, *c-Myc*, *CyclinD1*, *FGF20*) in HEK293 cells and HT29 cells. (C) DY (30 μg ml^−1^) down-regulated Wnt targeted genes levels (*Axin2* or *c-Myc*) in RKO and HCT116 cells. ^#^*P* < 0.05, *vs.* control group.

**Fig. 3:**
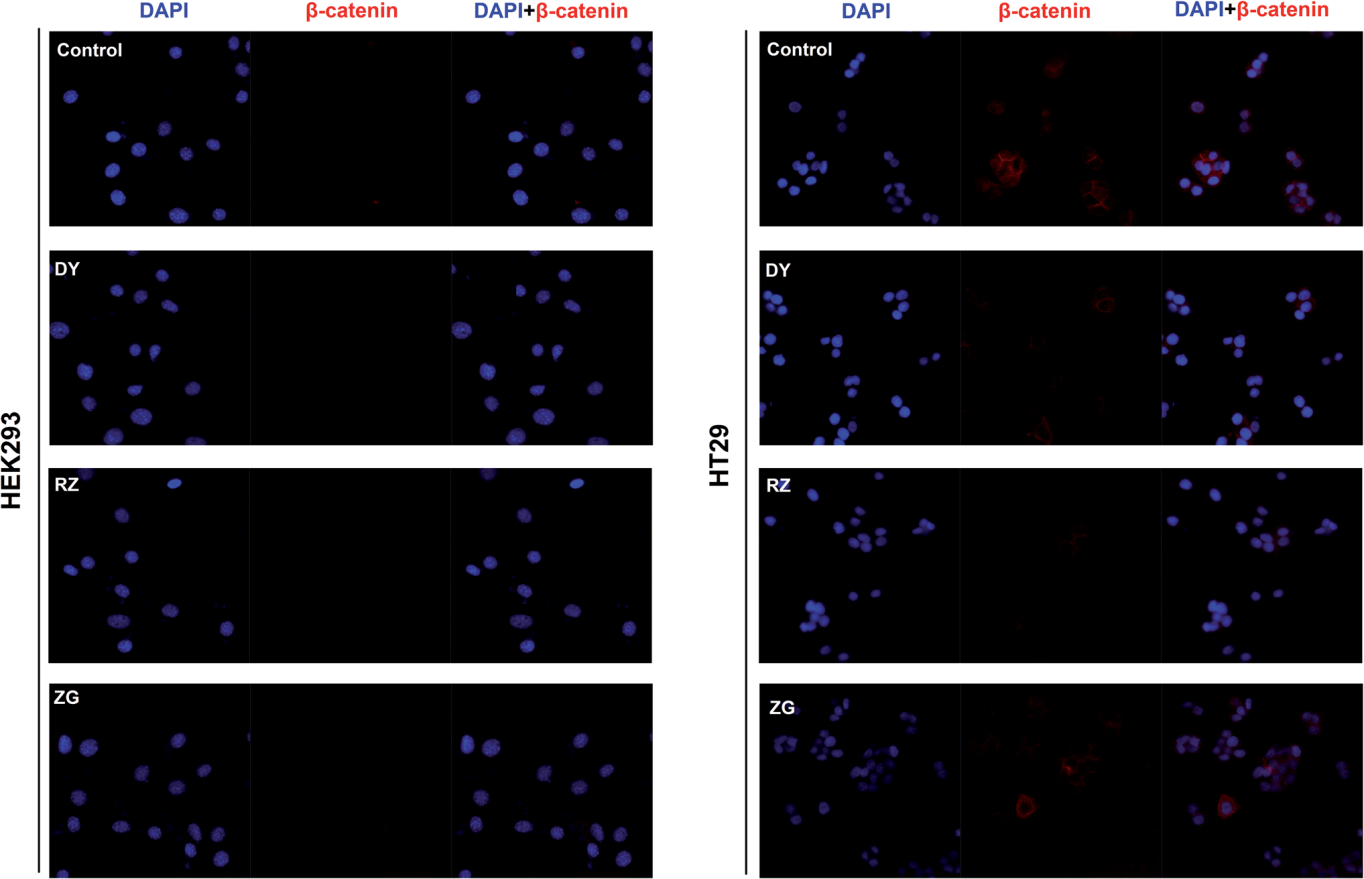
Effects of DY, RZ and ZG on β-catenin levels in the cytoplasm and nucleus of HEK293 and HT29 cells. Immunofluorescence assays were conducted to evaluate the influences of DY (30 μg ml^−1^), RZ (40 μg ml^−1^) and ZG (30 μg ml^−1^) on the β-catenin in HEK293 and HT29 cell lines. The nucleus was stained by DAPI, and subcellular localization of β-catenin (red) was detected by anti-β-catenin.

To further confirm the inhibitory effects of DY, RZ and ZG on the Wnt/β-catenin pathway, we studied their influences on the key proteins or genes of the canonical Wnt signaling that's associated with CRC. As reported, most initiation and development of CRC have resulted from the hyper-activation of Wnt pathway with genetic mutation of β-catenin.^20^ Through binding the transcription factors TCF and LEF, β-catenin would trigger the expression of Wnt target genes such as *Axin2*,^21^*CCK1*,^22,23^*c-Myc*,^6^*CyclinD1* (ref. 24) and *FGF20* (ref. 25) that are highly involved in the progression of colorectal cancer. Therefore, the levels of protein β-catenin and genes including *Axin2*, *CCK1*, *c-Myc*, *CyclinD1* and *FGF20* were further evaluated after DY, RZ and ZG treatments in this study. Western blot results suggest that, except for ZG, DY and RZ significantly down-regulated the amount of cytoplasmic β-catenin protein (Fig. 2A and s2A†). Meanwhile, to investigate whether DY and its constituents were able to promote the nuclear translocation of β-catenin or not, the immunofluorescence assay was conducted. Results show that, though β-catenin was not observed to be successfully transferred into the nucleus, the amounts of which in the cytoplasm were obviously decreased after DY and RZ treatments (Fig. 3). The reason why the nucleus translocation was not obvious as expected was possible due to the levels of β-catenin in the nucleus were so low when compared to that in the cytoplasm after nucleus translocation that the fluorescent signals were too weak to be detected by the confocal microscope. As the above findings were basically consistent, the Wnt-inhibiting activity of DY and ZG were further confirmed. For the qPCR experiment, the expression levels of GAPDH in different groups were first evaluated. Results indicate that GAPDH expression levels remained consistent at different experimental conditions (Fig. s3†). Based on this, ΔΔ*C*_t_ approach was then used to analyze the effects of medications on the levels of Wnt target genes. Data show that the mRNA levels of *Axin2*, *DKK1*, *c-Myc*, *CyclinD1* and *FGF20* were dramatically down-regulated by DY (Fig. 2A), which were also observed on RZ and ZG, except for gene *CyclinD1*.

### 3.2 DY, RZ and ZG inhibited the Wnt/β-catenin pathway in CRC cell

**Fig. 4:**
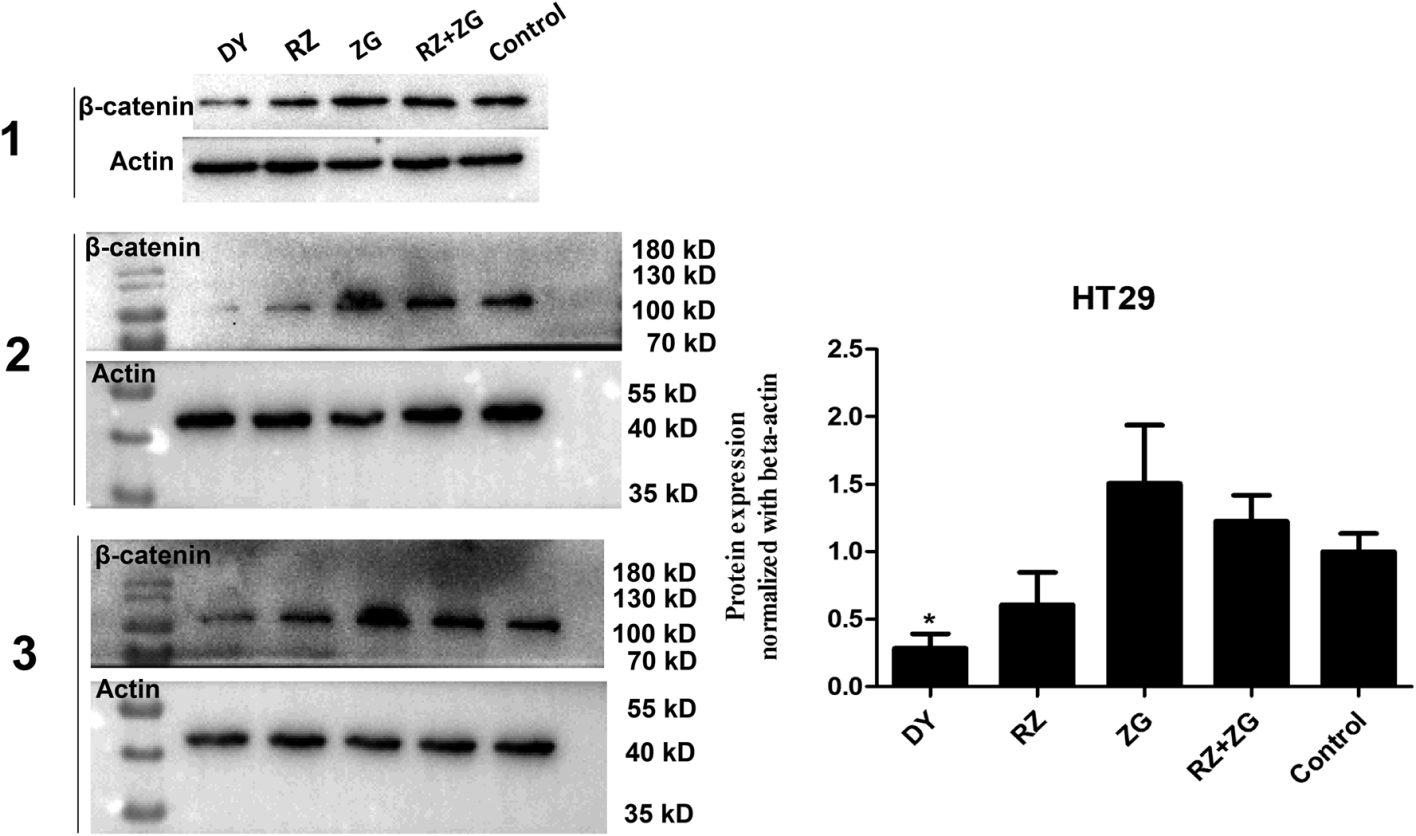
Effects of RZ, ZG and their combination on regulating β-catenin protein levels in the cytoplasm of HT29 cells. Left figure shows the blots of β-catenin and actin in HT29 cells from three independent experiments and their quantification results are showed in right diagram.

The activation of Wnt/β-catenin pathway is closely related to the incidence of colorectal cancer.^2^ Based on the inhibitory effects of DY, RZ and ZG on the Wnt signaling pathway in HEK293 cells, we further studied their influences on the Wnt pathway in a human colorectal cancer cell line, HT29. And to guarantee the objectivity of the results, the concentrations of DY extract and its fractions were determined based on the criteria proposed in HEK293 cell assays. Results of western blot assay show that only DY and RZ notably down-regulated the amounts of β-catenin protein in the cytoplasm of HT29 cells (Fig. 2B and s2B†). And Fig. 3 shows that DY and RZ effectively decreased β-catenin in the cytoplasm of HT29 cells, which validated the above findings. To further investigate the interactive inhibiting effects of RZ and ZG on Wnt signaling, HT29 cells were then solely or conjunctively treated with RZ and ZG at corresponding concentrations in 30 μg ml^−1^ DY. Data show that RZ deceased while ZG increased β-catenin level in the cytoplasm, and they didn't synergistically enhance the inhibitory effects for each other (Fig. 4). In terms of qPCR, only DY effectively inhibited two targeted genes levels of Wnt pathway, namely, *c-Myc* and *CyclinD1* (Fig. 2B). RZ merely down-regulated the levels of *c-Myc*. And in terms of ZG, no any inhibitory effects had been observed. On the contrary, it even significantly up-regulated *CyclinD1* expression. As *CyclinD1* plays an important role in promoting cell proliferation,^26^ which may be the potential factor of greatly limiting its Wnt-inhibiting activity. The above findings were not fully consistent to that in HEK293 cells. In fact, HT29 cell is different from the HEK293 cell at gene levels. In HT29 cells, the Wnt/β-catenin signaling pathway has been innately and aberrantly activated by the mutation of *APC*^27^ and Wnt-related oncogenes especially *c-Myc*^28^ and *CyclinD1* (ref. 29) are over-expressed. Therefore, without Wnt3a, DY and RZ significantly down-regulated *c-Myc* and *CyclinD1*, verifying the inhibitory activities of DY and RZ on Wnt signaling as well. In addition to HT29 cells, another two colorectal cancer cell lines (RKO and HCT116) were studied to confirm the Wnt-inhibiting effects of the indicated medications on CRC cells. Based on Fig. 2C, we found that DY significantly decreased *Axin2* levels in HCT116 cells and showed a tendency of down-regulating *Axin2* and *c-Myvc* in RKO cells, which further verified its inhibitory effects on Wnt signaling pathway of CRC cells. Other genes were not significantly influenced. Western blot results are not available here, because no blots of β-catenin were observed in the two CRC cell lines.

### 3.3 The transcriptomics study of DY and its main components on the HT29 cell

Transcriptomic profiling study is a preferred strategy to find out the molecular mechanisms of certain biological events based on a holistic view. We took use of this high-throughput technology to systematically explore the underlying mechanisms accounting for the inhibitory effects of DY and its main components on the Wnt pathway of CRC cells-HT29 cell. The RNA-seq data can be referred to the link https://figshare.com/s/c20cae72a30e6551c962, including function annotation, GO analysis, GO tree analysis, KEGG pathway analysis and so on. Table s3 and Fig. s4† showed that, except for ZG, DY and RZ could induce a great number of DEGs, demonstrating that DY and RZ prominently influenced the biological profiles of HT29 cells. Next, we conducted Gene Ontology (GO) analysis to classify the DEGs. Through comparing with the enrichment results of the GO secondary functions at the all-gene background, we found that DY, RZ and ZG induced significant changes of genes involved in different functional branches (Fig. s5†). The data Fig. s6† showed that the DEGs in DY, RZ and ZG groups were all mainly involved in cell proliferation, cell cycle and cell metabolism. For instance, the genes enriched in Biological Process (BP) were related to small molecule metabolic process, neurotropic TRK receptor signaling pathway, wound healing and synaptic transmission. Similar phenomenon were observed in Cellular Component (CC) and Molecular Function (MF) as well. Based on the fact that the aberrant regulation of cell growth and cell death is closely associated with the initiation and progression of carcinomas, the GO results may indicate DY, RZ and ZG exert therapeutic potency towards HT29-based CRC.

In addition to GO analysis, Kyoto Encyclopedia of Genes and Genomes (KEGG) analysis was further carried out to explore the potential functions of the DEGs. Fig. s7† shows the pathways enriched with different DEGs. Results indicated that the DEGs contributed by DY and its main components were from multiple biological processes, including cellular processes, environment information processing, genetic information processing, human diseases, metabolism and organismal systems. And most of DEGs were enriched in the pathways associated with human diseases and metabolism. In the DY group, human diseases-DEGs were mainly distributed in pathways such as pathways in cancer, chemical carcinogenesis, herpes simplex infection and influenza A infection, and the metabolism-related DEGs were enriched in other pathways, including pentose phosphate pathway, steroid hormone biosynthesis and metabolism of xenobiotic by cytochrome 450 and so on. In the RZ group, in addition to the overlapped pathways in the DY-treated group, it was also able to prominently regulate the genes related to viral and renal carcinomas. Furthermore, genes involved in diseases such Type II diabetes mellitus, tuberculosis, *Vibrio cholerae* infection, toxoplasmosis, HTLV-I infection and hepatitis C were obviously regulated by RZ as well. As reported, tannins exerted anti-proliferative activities to many cancer cell lines such as MCF-7, Caco-2 and DU145,^30^ and it could effectively treat kinds of human diseases including inflammation, Type II diabetes mellitus and leukemia.^31–33^ Hence, the KEGG results basically conform to the reported facts. In the ZG group, all the DEGs were enriched in metabolic pathways, namely, ABC transporters, purine metabolism and drug metabolism-other enzymes. Furthermore, the above findings were confirmed using statistical analysis of pathway enrichment (Fig. s8†).

### 3.4 The analysis of DEGs in the Wnt/β-catenin pathway and its cross-talking pathways

**Fig. 5:**
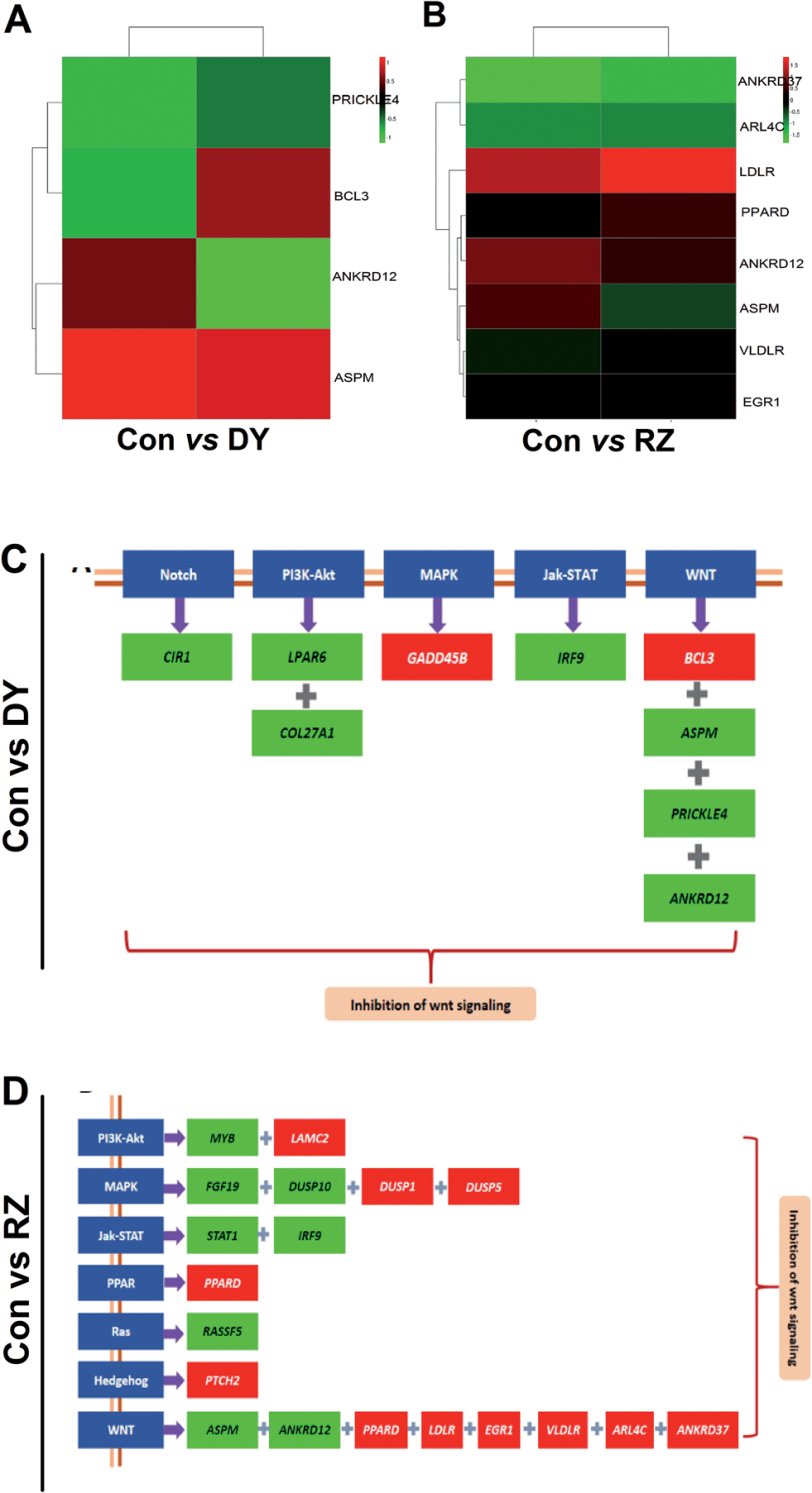
Effects of DY and RZ on Wnt pathway-related genes. (A) and (B) represent Heatmaps of DEGs in the Wnt/β-catenin signaling pathway of HT29 cells. DEGs are annotated in the Wnt pathway and respectively induced by DY and RZ. (C) and (D) show the DEGs in the Wnt and its cross-talking pathways of HT29 cells. The names of the indicated pathways are shown in blue boxes, while the down-regulated and up-regulated genes are presented in green and red boxes, respectively.

Based on the findings that DY and RZ significantly regulated genes involved in cancers, we analyzed the DEGs on the cancerous pathways, especially the Wnt/β-catenin signaling pathway and its cross-talking pathways including Notch, PI3K-Akt, MAPK, Jak-STAT, PPAR, Ras and Hedgehog.^34^ In terms of Wnt signaling pathway, DY and RZ only significantly regulated a few of genes, while no DEGs were observed in the ZG group (Fig. 5A/B). For the Wnt-related DEGs, DY up-regulated *BCL3* and down-regulated *PRICKLE4*, *ANKRD12* and *ASPM*, while RZ up-regulated *ARL4C*, *EGR1*, *PPARD*, *VLDLR*, *LDLR* and down-regulated *ANKRD12* and *ASPM* (Fig. 5C). Moreover, DY and RZ also induced DEGs in the cross-talking pathways of Wnt/β-catenin signaling pathway. Results in Fig. 5D show that, in the DY-treated group, mRNA levels of *CIR1*, *LPAR6*, *COL27A1* and *IRF9* in Notch, PI3K-Akt and Jak-STAT pathways were down-regulated, while *GADD458* in MAPK was up-regulated. In the RZ-treated group, *MYB* (PI3K-Akt), *FGF19* & *DUSP10* (MAPK), *STAT1* & *IRF9* (Jak-STAT) and *RASSFS* (Ras) were down-regulated, while *LAMC2* (PI3K-Akt), *DUSP1* & *DUSP5* (MAPK), *PPARD* (PPAR) and *PTCH2* (Hedgehog) were up-regulated. As reported, the above genes played important roles in the hyperactivation of Wnt pathway and they were also found generally over-expressed in the Wnt-activated CRC.^35–41^ Therefore, current findings suggest that the inhibitory activities of DY and RZ towards the Wnt/β-catenin signaling pathway might be relevant to their down-regulation of the indicated genes, which should be further confirmed. Taken together, DY and RZ can effectively down-regulate the key protein and genes levels in the Wnt signaling pathway. As colon cancer is highly associated with the abnormal activation of Wnt pathway and DY and RZ were able to significantly inhibit Wnt pathway, we hypothesize that they exhibit anti-CRC potentials, which would be further verified in our future work.

## 4. Conclusion

In conclusion, the inhibitory effects of an aqueous extract of DY on the Wnt/β-catenin signaling pathway of HEK293 is first reported. The study of bioactive constituents demonstrates that RZ and ZG of DY played important roles in inhibiting Wnt pathway, while their representative constituents were not the active as expected. The active compounds in DY are still under investigation in our lab. DY and RZ also could significantly inhibit the Wnt pathway in CRC cell line, HT29, which might be resulted from their down-regulation of Wnt-related genes such as *LPAR6*, *PRICKLE4*, *ASPM*, *MYB*, *FGF19* and *STAT1.* All in all, our findings suggest that DY and might be a good resource of Wnt inhibitors. As CRC is highly associated with the aberrant activation of Wnt pathway, DY is also possible to be developed as anti-CRC agents when supported by more pre-clinical and clinical evidence, which will be studied in the near future.

## Conflicts of interest

None declared.

## Supplementary Material

RA-008-C8RA00438B-s001
