# Supplementary material for: Aqueous extract of Sanguisorba officinalis blocks the Wnt/β-catenin signaling pathway in colorectal cancer cells
Source: RSC Adv. 2018 Mar 13;8(19):10197–206. doi: 10.1039/c8ra00438b (PMC9078833; doi:10.1039/c8ra00438b)
Supplement: RA-008-C8RA00438B-s001 [file RA-008-C8RA00438B-s001.pdf]

Electronic Supplementary Material (ESI) for RSC Advances.
This journal is © The Royal Society of Chemistry 2018

Supplementary materials

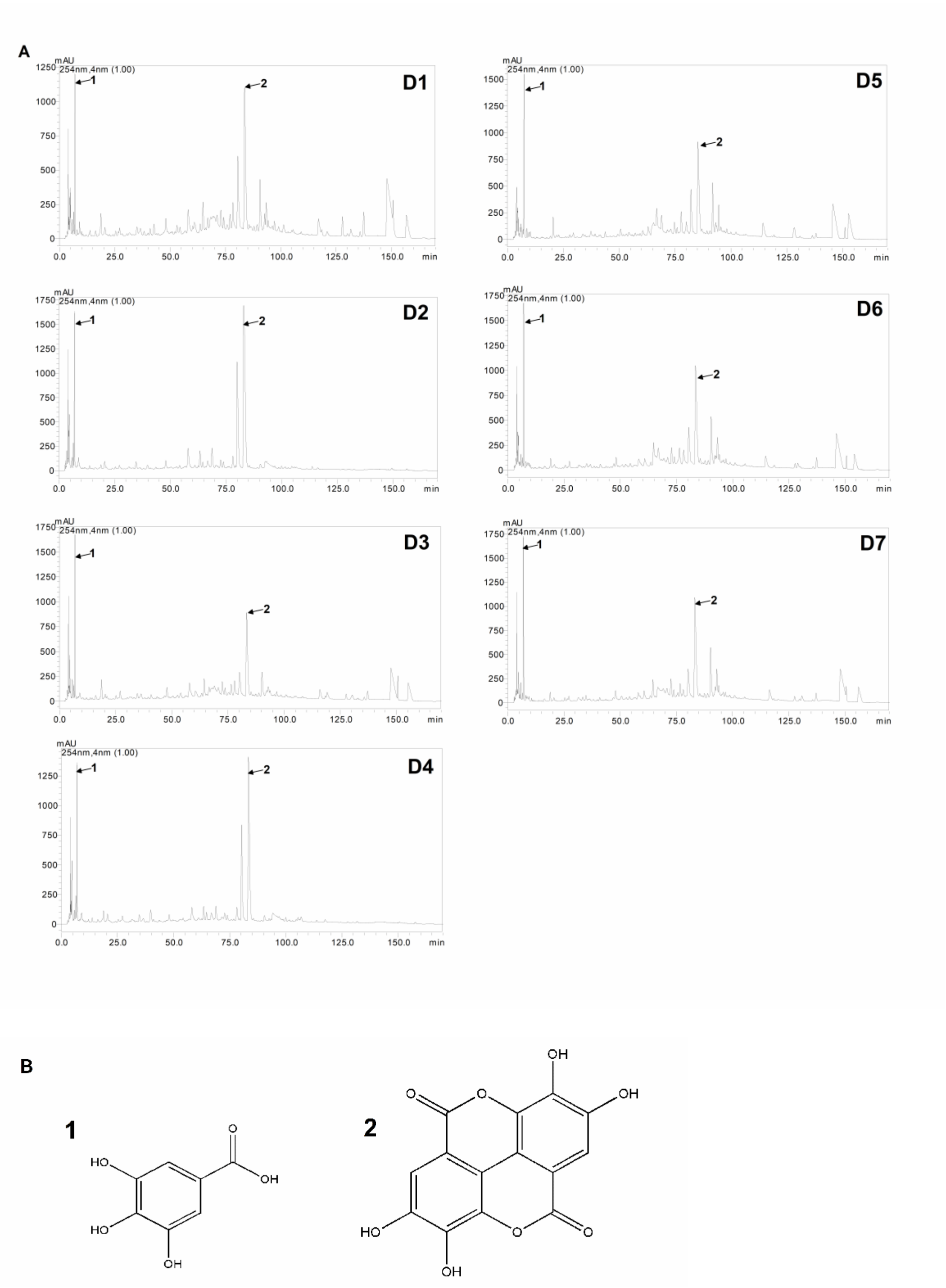

Figure. s1. **Constituent analysis of the aqueous extracts of 7 batches of DY (D1-D7).** (A) HPLC chromatograms of D1-D7. The information of D1-D7 was showed in **Table s1**. (B) The structures of chemical 1 and 2 marked in (A).

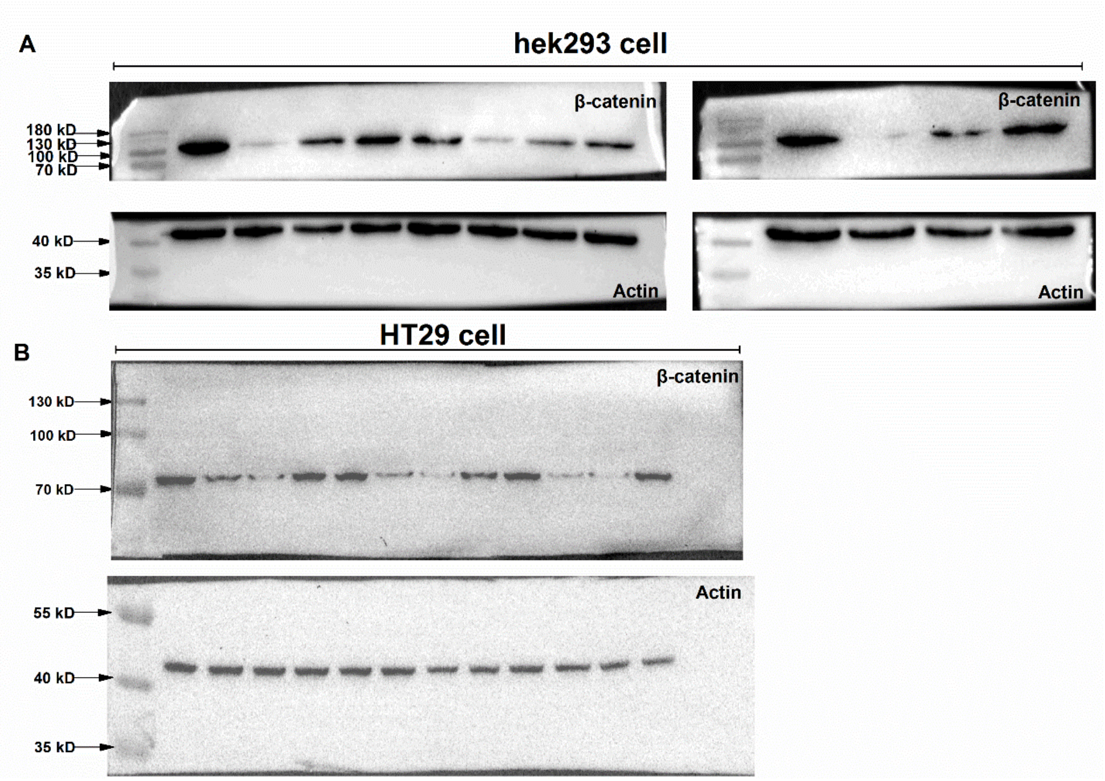

Figure S2. **Western blots with molecular size markers for the indicated proteins in the manuscript.** (A) and (B) are the blots of β-catenin and actin in HEK293 and HT29 cells from three replicated experiments, respectively. The blots of each protein in the control, DY, RZ and ZG groups are presented from left to right in each cell line.

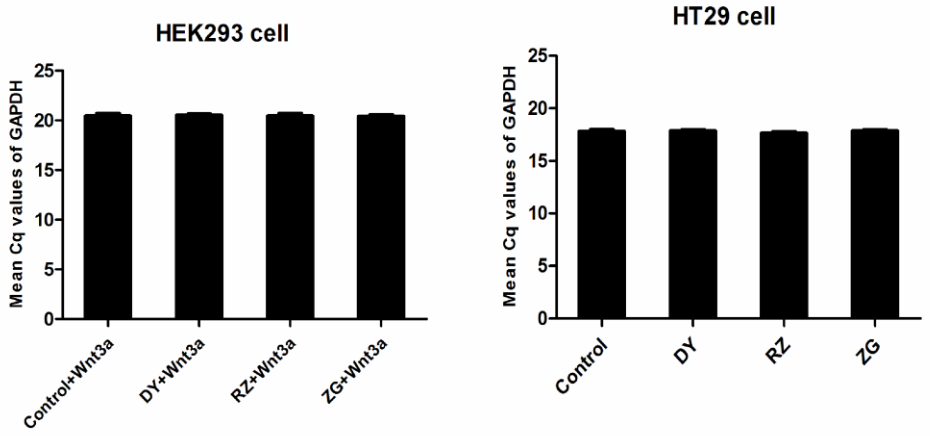

Figure s3. **Mean Cq values of GAPDH in HEK293 and HT29 cells after being treated with different medications for 24 h.** *P<0.05, indicating significant difference.

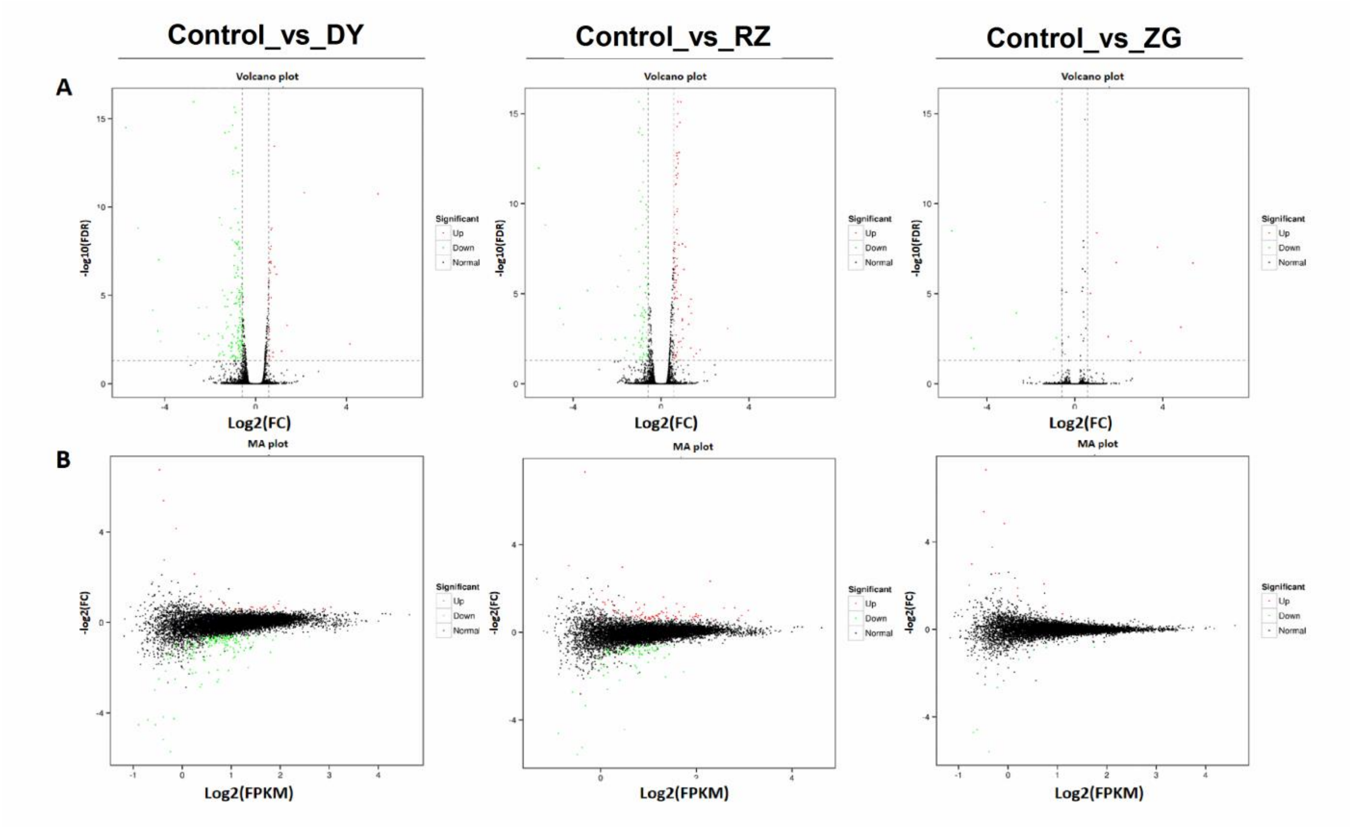

Figure s4. **Integral distribution of the DEGs of HT29 cells.** (A) and (B) are volcano plots and MA graphs of DEGs induced by DY (30 μg/ml), RZ (40 μg/ml) and ZG (30 μg/ml). FC, fold change; FDR, False Discovery Rate; FPKM, Fragment Per Kilobase of exon model per Million mapped reads. The up-regulated, down-regulated and normal genes are respectively plotted as red, green and black dots. DESeq was used to perform the DEGs analysis.

	Biological Process	KS	Cellular Component	KS	Molecular Function	KS
**Con_vs_DY**	small molecule metabolic process	1.20E-14	cytoplasmic part	2.30E-27	protein kinase binding	3.40E-17
	neurotrophin TRK receptor signaling pathway	3.00E-14	intracellular membrane-bounded organelle	7.00E-22	ATP binding	2.20E-15
	synaptic transmission	9.50E-14	nucleolus	4.80E-17	cytoskeletal protein binding	1.60E-12
	positive regulation of transcription from RNA polymerase II promoter	2.10E-13	intracellular organelle	9.60E-16	protein binding	3.70E-10
	protein autophosphorylation	7.10E-13	centrosome	2.10E-15	magnesium ion binding	4.00E-10
	epidermal growth factor receptor signaling pathway	1.50E-12	neuron projection	2.20E-15	transcription coactivator activity	2.90E-09
	wound healing	1.20E-10	chromosome, centromeric region	4.90E-13	phosphatidylinositol binding	1.90E-08
	positive regulation of transcription, DNA-templated	4.50E-10	cell projection	1.00E-12	activin binding	4.70E-08
	positive regulation of neuron projection development	6.30E-10	microtubule organizing center	3.10E-12	SMAD binding	1.10E-07
	actin cytoskeleton reorganization	4.40E-09	cell projection part	1.50E-11	anion binding	1.20E-07
**Con_vs_RZ**	small molecule metabolic process	2.90E-15	cytoplasmic part	7.30E-27	protein kinase binding	2.40E-17
	neurotrophin TRK receptor signaling pathway	1.80E-14	intracellular membrane-bounded organelle	2.50E-21	ATP binding	1.30E-15
	positive regulation of transcription from RNA polymerase II promoter	6.40E-14	nucleolus	5.50E-17	cytoskeletal protein binding	1.80E-12
	protein autophosphorylation	2.50E-13	intracellular organelle	8.60E-16	magnesium ion binding	2.30E-10
	synaptic transmission	3.50E-13	neuron projection	2.70E-15	protein binding	1.60E-09
	epidermal growth factor receptor signaling pathway	4.00E-13	centrosome	7.40E-15	transcription coactivator activity	2.90E-09
	positive regulation of transcription, DNA-templated	4.10E-11	chromosome, centromeric region	2.10E-13	phosphatidylinositol binding	6.20E-09
	wound healing	1.30E-10	cell projection	3.90E-12	activin binding	4.80E-08
	positive regulation of neuron projection development	7.10E-10	microtubule organizing center	1.70E-11	SMAD binding	1.10E-07
	nervous system development	2.00E-09	cell projection part	3.10E-11	SH3 domain binding	1.20E-07
**Con_vs_ZG**	neurotrophin TRK receptor signaling pathway	1.50E-14	cytoplasmic part	1.20E-24	protein kinase binding	2.00E-17
	positive regulation of transcription from RNA polymerase II promoter	1.00E-13	intracellular membrane-bounded organelle	2.00E-17	ATP binding	7.80E-16
	synaptic transmission	1.60E-13	nucleolus	8.20E-17	cytoskeletal protein binding	6.00E-12
	protein autophosphorylation	3.70E-13	neuron projection	1.50E-15	magnesium ion binding	2.40E-10
	small molecule metabolic process	5.00E-13	intracellular organelle	1.80E-15	protein binding	2.70E-10
	epidermal growth factor receptor signaling pathway	7.90E-13	cell projection	1.20E-14	transcription coactivator activity	1.90E-09
	wound healing	7.20E-11	centrosome	1.70E-14	phosphatidylinositol binding	1.30E-08
	positive regulation of neuron projection development	4.20E-10	microtubule organizing center	1.40E-12	activin binding	3.70E-08
	positive regulation of transcription, DNA-templated	7.40E-10	chromosome, centromeric region	2.20E-12	SMAD binding	8.70E-08
	actin cytoskeleton reorganization	3.00E-09	cell junction	4.10E-11	SH3 domain binding	1.70E-07

***Figure s5.* The GO enrichment analysis of DEGs.** The top 10 secondary functions of the 3 GO items (BP, CC and MF) enriched with DEGs in DY, RZ and ZG groups were listed. KS represented the significant difference of the GO enrichment. The significance of the statistics difference increased with KS value. T01-T04 respectively represented the control, DY, RZ and ZG groups.

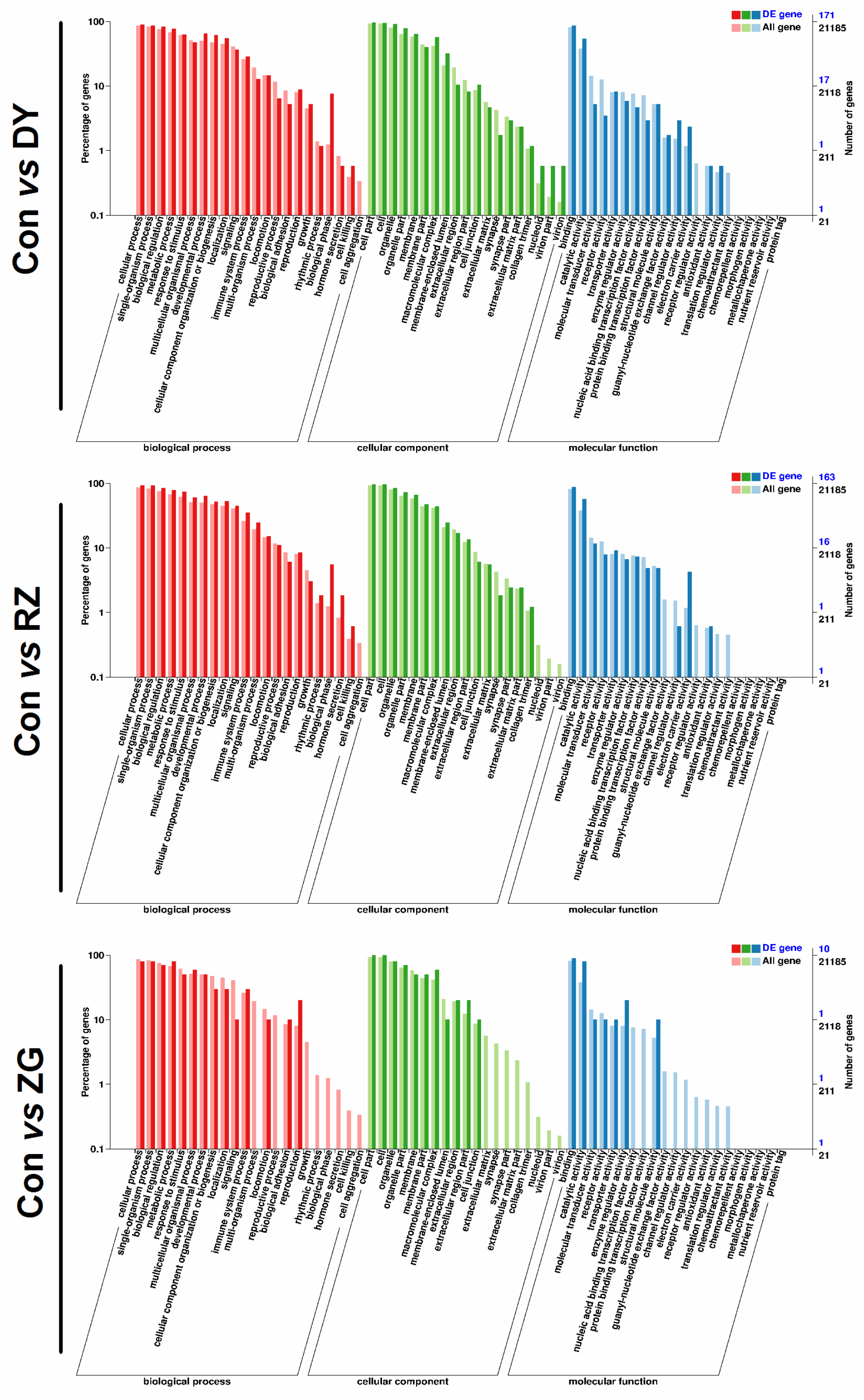

Figure s6. **GO classification of the DEGs.** X-coordinate shows the classifications (BP, CC, MF) of the GO items, and the percentage and numbers of genes are respectively presented on the left and right sides of y-coordinate. The secondary functions showing significant differences (DEGs vs All gene) are regarded as the potential functions

accounting for the production of DEGs. topGO was applied to conduct the GO enrichment analysis.

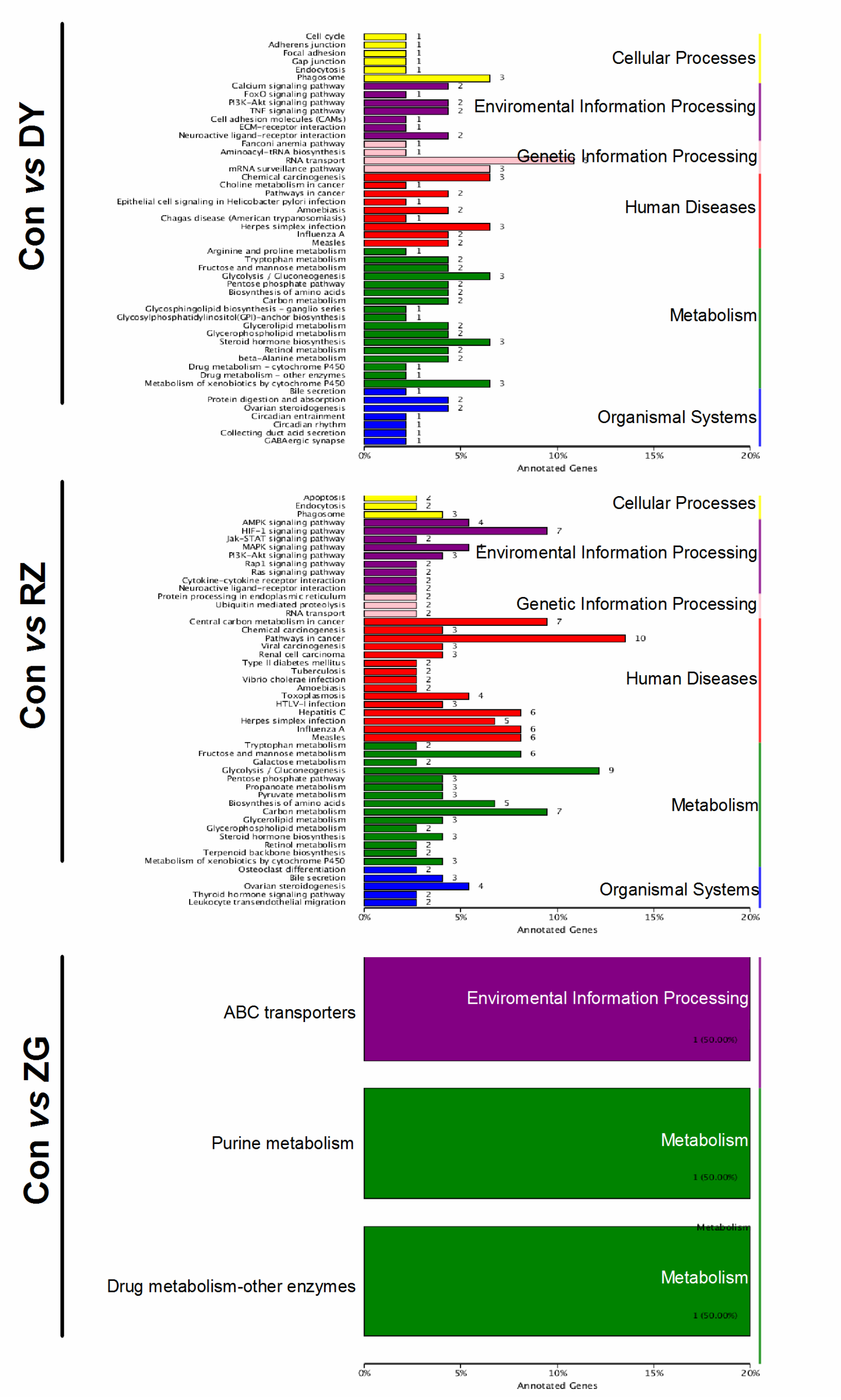

Figure s7. **KEGG annotation of the DEGs.** X-coordinate shows the number and percentage of the genes annotated in the indicated pathway, while the y-coordinate presents the names of the annotated pathways. KEGG database

(http://www.genome.jp/kegg/) was referred to conduct the KEGG annotation analysis.

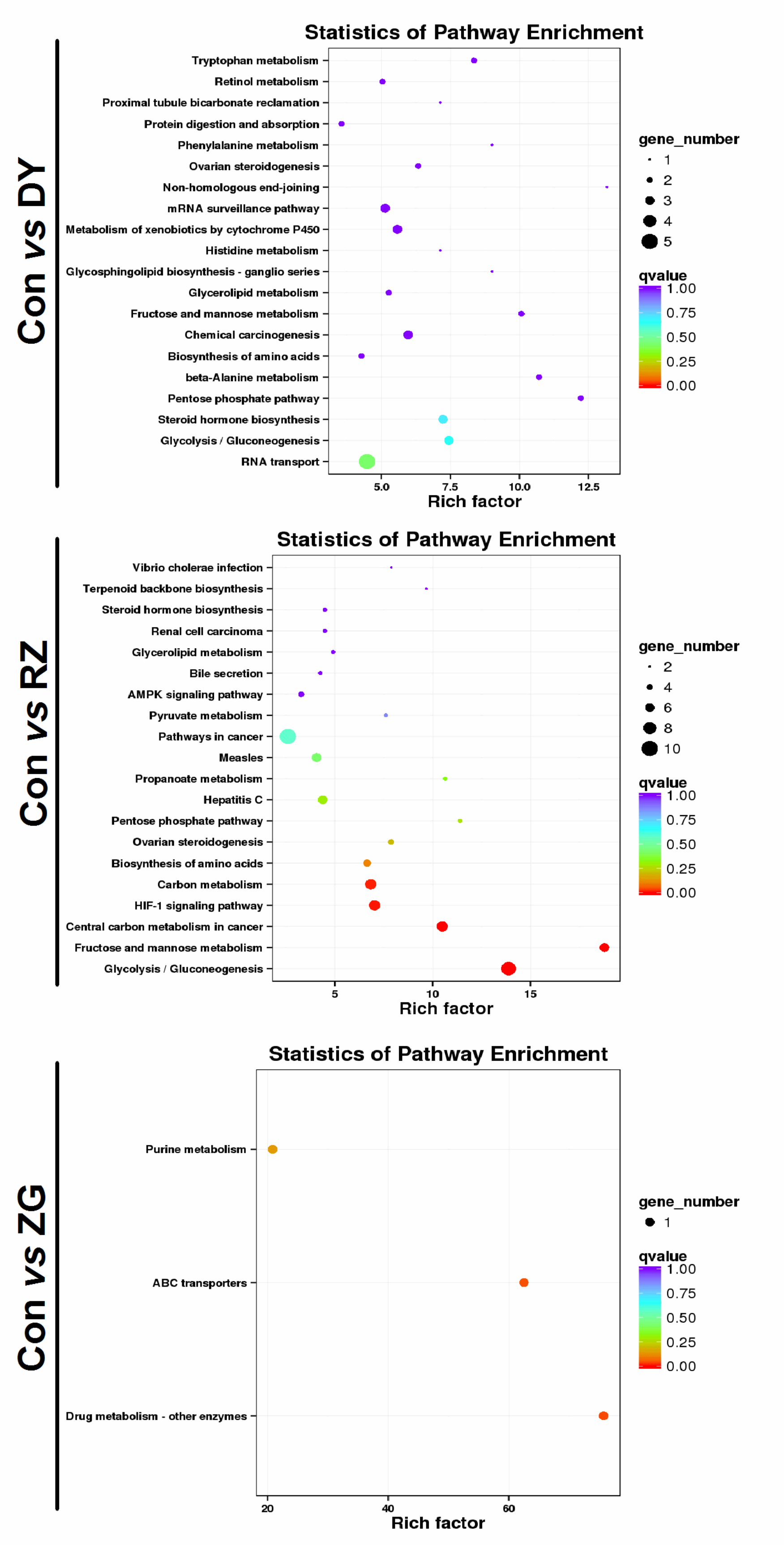

Figure s8. **The KEGG enrichment analysis of DEGs.** X-coordinate showed the Rich

Factor (RF), and the enrichment extent of DEGs in certain pathways enhances with the RF values. Per circle represented a KEGG pathway. The colors and sizes of the circles respectively indicated the P values and gene numbers. The reliability of the KEGG enrichment and the enriched gene numbers were increased with the P value and circle size, respectively.

Table s1

The information of the 7 batches of *sanguisorba officinalis*

Herb No.	Specimen No.	Original place
D1	DY-1-20150403	Jiangsu
D2	DY-2-20151011	Heilongjiang
D3	DY-3-20151011	Hebei
D4	DY-4-20151011	Guangdong
D5	DY-5-20160201	Shanxi
D6	DY-6-20160201	Fuzhou
D7	DY-7-20160201	Hebei

Table s2

The IC50 values of 7 batches of sanguisorba officinalis on the Wnt/β-catenin pathways

Herb No.	IC_{50} (μg/ml)
D1	2.20 ±0.74
D2	26.80 ±8.67
D3	16.81 ±2.75
D4	41.93 ±21.39
D5	20.50 ±8.18
D6	12.85 ±2.44
D7	24.42 ±7.44

IC_{50} Values were expressed as mean ±SD

Table s3

The statistic results of the DEGs in HT29 cells

DEG set	DEG Number	Up-regulated	Down-regulated
T01_vs_T02	209	32	177
T01_vs_T03	190	94	96
T01_vs_T04	20	11	9
